# Bilateral Neonatal Adrenal Hemorrhage Associated With Severe Maternal COVID-19 Infection

**DOI:** 10.7759/cureus.20007

**Published:** 2021-11-29

**Authors:** Marcio José Concepción-Zavaleta, Sofia Pilar Ildefonso-Najarro, Esteban Plasencia-Dueñas, Julia Cristina Coronado Arroyo, Francisca Elena Zavaleta-Gutiérrez, Luis Concepción-Urteaga, Frederick Massucco Revoredo, Anthony Ramos-Yataco, Kelly Meza

**Affiliations:** 1 Endocrinology, Clínica Stella Maris, Lima, PER; 2 Endocrinology, Diabetes and Metabolism, Hospital Nacional Guillermo Almenara, Lima, PER; 3 Obstetrics and Gynecology, Clínica Vesalio, Lima, PER; 4 Pediatrics and Neonatology, Hospital Belén de Trujillo, Trujillo, PER; 5 Internal Medicine, Hospital Regional Docente de Trujillo, Trujillo, PER; 6 Endocrinology, Hospital Nacional Guillermo Almenara Irigoyen, Lima, PER; 7 Internal Medicine, Hospital Ricardo Cruzado Rivarola, Nasca, PER; 8 Pediatric Nephrology, Weill Cornell Medicine, New York, USA

**Keywords:** fetal distress, adrenal haemorrhage, new-born, covid-19 outbreak, adrenal glands

## Abstract

Adrenal hemorrhage is the most common cause of adrenal mass in newborns. We present a case of a full-term male, born by cesarean section due to acute fetal distress from a mother with severe coronavirus disease 2019 (COVID-19) infection. He was diagnosed with hypoxic-ischemic encephalopathy, multifactorial shock, and early neonatal sepsis. On the seventh day of hospitalization, hemoglobin dropped and thus blood transfusion was required, and abdominal ultrasound showed bilateral adrenal hemorrhage. He developed relative adrenal insufficiency without either hemodynamic instability or electrolyte imbalances. The use of parenteral corticosteroids was not required. Follow-up ultrasonography and adrenal axis laboratory examination revealed complete resolution of adrenal hemorrhage. Neonatal adrenal hemorrhage has a wide variety of clinical manifestations. Ultrasound is preferred for both initial screening and follow-up evaluation. Adrenal insufficiency occurs rarely in neonatal adrenal hemorrhage. Treatment is usually conservative. We emphasize the importance of a timely diagnosis and clinical follow-up of adrenal hemorrhage in neonates with fetal distress born from mothers with severe COVID-19.

## Introduction

Adrenal glands are relatively larger and more vascular early in life than in adulthood [1-2]. It generates greater vulnerability to mechanical compression and greater sensitivity to changes in venous pressure and redistribution of blood flow in cases of hypoxia [2]. Adrenal hemorrhage (AH) is more frequent in the neonatal period, it is currently considered the most common cause of adrenal mass in this age group [3-4]. Its incidence ranges between 1.7 and 3 cases per 1000 live births [1,3,5]; however, this value could be higher (16-29 per 1000 live births) since they are mostly asymptomatic [6].

AH is frequently seen in male neonates, 90% of cases are unilateral [1]. Clinical manifestations are variable although they are often absent, when symptomatic patients present with jaundice, ischemia, palpable abdominal mass, and anemia, in severe cases adrenal insufficiency (AI), hypovolemic shock, and even death may occur [2,4-5]. Development of adrenal insufficiency is rare since 10% of functional adrenal tissue produces enough amount of cortisol to meet the body´s needs [3,5]. It explains why most patients do not require treatment, and the process is usually self-limited with complete resolution within 20 and 165 days of life [2-3]. Even in cases where adrenal insufficiency developed and treatment is needed, complete resolution is achieved [3].

Adrenal hemorrhage in neonates has several risk factors, such as hypoxia, birth asphyxia, coagulation disorders, traumatic delivery, bacterial sepsis, and coronavirus disease 2019 (COVID-19) might be a new risk factor [2]. COVID-19 increases the rate of premature birth, fetal distress, and neonatal respiratory distress syndrome, these factors may contribute to the development of AH [7].

In this manuscript, we present the case of a newborn with a bilateral adrenal hemorrhage who developed relative adrenal insufficiency and whose mother had a severe COVID-19 infection.

## Case presentation

A male infant was born at 39 weeks of gestation from a 31-year-old mother with three antenatal check-ups during pregnancy. She presented to a primary care clinic with shortness of breath; her oxygen saturation was 90% on room exam. She tested immunoglobulin M (IgM)/IgG positive for severe acute respiratory syndrome coronavirus 2 (SARS-CoV-2) in an immunochromatography test; severe COVID-19 was diagnosed. Fetal ultrasonography revealed signs of fetal distress, thus an emergency cesarean section was performed. The neonate had adequate weight and height, Apgar scores were 3-6-7 at the first, fifth, and tenth minutes, respectively. He had marked respiratory distress nine hours after delivery. The patient was intubated and referred to a higher-level hospital.

On admission patient´s vital signs were as follow: blood pressure 75/49 mmHg, heart rate 130 beats per minute (bpm); respiratory rate 70 cycles per minute, body temperature 36.7 Celsius degrees, and oxygen saturation 96%, on oxygen fraction of 40% with a peak inspiratory pressure in 16 cm of water and positive pressure at the end of expiration (PEEP) in 5 cm of water. On physical exam, the patient was found stuporous, pale, with distal coldness, capillary filling equal to 3 seconds, perioral cyanosis, and presence of blood in the orogastric tube. On respiratory exam, an irregular respiratory pattern was observed and decreased breath sound bilaterally. Abdominal sounds were diminished and signs of inflammation around the umbilical stump were found. Pupils were miotic; there was little response to stimuli, hypotonia, no sucking reflex, and neither palmar nor plantar grasp were found.

Laboratory examination, blood cultures, arterial blood gas analysis (ABGA) (Table 1), and RT-PCR (real-time reverse transcription-polymerase chain reaction) for SARS-CoV-2(severe acute respiratory syndrome coronavirus 2) infection were obtained. ABGA revealed metabolic acidosis, hypoglycemia, hyponatremia, and hypocalcemia. RT-PCR for SARS-CoV-2 was negative. Due to laboratory findings and physical exam hypoxic-ischemic encephalopathy and multifactorial shock, early neonatal sepsis was diagnosed. He was started on 3% sodium chloride, 10% dextrose, dopamine 8 ug/Kg/min, dobutamine 8 ug/Kg/min, sodium bicarbonate, calcium gluconate, phytomenadione, fresh frozen plasma, meropenem 80 mg intravenous every eight hours, and vancomycin 60 mg intravenous every eight due to abrupt clinical deterioration.

**Table 1:** Blood tests obtained during hospitalization RV: reference value, PT: prothrombin time, aPTT: activated partial thromboplastin time, INR: international normalized ratio, AST: aspartate aminotransferase, GGTP: gamma-glutamyl transpeptidase, TG: triglycerides, TSH: thyroid-stimulating hormone, T4: thyroxine, ACTH: adrenocorticotropin, PTH: parathormone, RT-PCR: real-time reverse transcription-polymerase chain reaction, TORCH: toxoplasma, syphilis, rubella, cytomegalovirus, and herpes simplex virus

Parameter	Result
Complete blood count on admission	Leucocytes: 18,000 cells per microliter (segmented neutrophils: 71%, abastonades: 3%), hemoglobine: 17.1 g/dl, platelets: 51000 cells per microliter
Coagulation profile on admission	TP: 10.97 seconds, fibrinogen: 3.75, TTPa: 43.11 seconds, INR: 0.97
Liver profile on admission	AST: 26 UI/L, albumine: 4.1 g/dl, total bilirrubine: 8.74 g/dl, indirect bilirrubine: 8.04 g/dl, GGTP: 163 UI/L, ALP: 226 UI/L
Biochemical profile and electrolytes on admission	Glucose: 87 mg/dl, serum sodium: 132 mmol/L, serum potasium: 4.3 mmol/L, total calcium: 8 mg/dl, magnesium: 2.26 mg/dl and serum phosphorus: 5.3 mg/dl
Renal function on admission	Creatinine: 0.48 mg/dl, urea: 21.4 ng/dl
Lipidic profile	Total cholesterol: 147 mg/dl, LDL cholesterol: 72 mg/dl, HDL cholesterol: 27 mg/dl, TG: 172 mg/dl
Hormonal profile	TSH: 3.58 mUI/L (VR: 0.44-8.8 mUI/L), T4 libre: 1.45 ng/dl (VR: 0.48-2.32 ng/dl), ACTH: 52 pg/ml (VR: 25-100 pg/ml), cortisol basal: 5 ug/dl (VR: 5-25 ug/dl), PTH: 10 pg/ml (VR: 9-52 pg/ml)
Other ancillary exams	RT-PCR-SARS-CoV-2: negative, viral hepatitis profile: negative, Epstein Barr virus antibodies: negative, TORCH profile: negative, immunoglobulin dosage: normal and lymphocyte count CD4/CD8: normal. Neonatal screening negative

**Figure 1:**
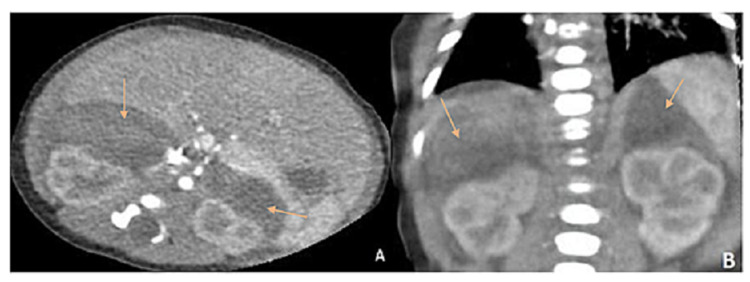
Findings of abdominal CT with contrast, showing bilateral adrenal hematomas Coronal (A) and sagittal (B) sections Arrows indicate bilateral adrenal hemorrhage

During hospitalization, the patient had subtle tonic seizures, a transfontanellar ultrasound showed signs of cerebral edema, therefore phenobarbital and subsequently levetiracetam were initiated due to a suboptimal response. On the seventh day of hospitalization, hemoglobin dropped up to 11 g/dl, which prompted blood transfusion. An abdominal ultrasound revealed on the right adrenal gland, a heterogeneous and hypoechoic encapsulated lesion, measuring 41 x 22 x 40 mm, with a volume of 21 ml; on the left adrenal region, another lesion with similar characteristics, measuring 40 x 17 x 30 mm, with a volume of 11 ml. AH was suspected thus abdominal contrast tomography was taken (Figure 1), which revealed a retroperitoneal hypodense collection on both adrenal glands, confirming the diagnosis of bilateral adrenal hemorrhage.

The patient was evaluated by the surgery team; no surgical management was recommended due to comorbidities and unaltered hemodynamic status. He was evaluated by the endocrinology team, which ordered a lab examination (Table 2). After his reassessment, he was diagnosed with relative adrenal insufficiency. Since there wasn’t hemodynamic instability or electrolyte imbalances, clinical monitoring and periodic ultrasound were recommended. During the three-month follow-up, the adrenal hemorrhage and relative adrenal insufficiency resolved (Table 2).

**Table 2:** Correlation between ultrasound findings, serum electrolytes, cortisol, and ACTH in the patient ACTH: adrenocorticotropin

	AT THE TIME OF DIAGNOSIS	1 MONTH LATER	3 MONTHS LATER
Abdominal ultrasound	Right adrenal hematoma (volume: 20 ml), Left adrenal hematoma (volume: 11 ml)	Right adrenal hematoma (volume: 14 ml), Left adrenal hematoma (volume: 8 ml)	Adrenal glands with normal characteristics, without observing collections dependent on them
Basal cortisol (ng/dl)	4.22	3.9	7.2
ACTH (pg/ml)	52	17.9	43
Serum sodium (mmol/l)	141.5	135.4	138.8
Serum potasium (mmol/l)	4.6	5.06	4.12

## Discussion

AH usually occurs in the neonatal period. Newborns usually have a larger size and increased vascularity of the adrenal glands, which make them vulnerable to mechanical compression and increased sensitivity to changes in venous pressure during delivery [6]. In addition, hypoxia may cause a redistribution of blood to the central nervous system [6-8]. As a result, congestion and endothelial damage may lead to AH [9].

AH in neonates is considered a rare condition, which accounts for about 0.2% to 0.55% of adrenal mass cases [10-11]. It may actually have a higher incidence since it is mostly asymptomatic. AH is usually an incidental finding. It is generally unilateral, affecting the right adrenal gland in 70% of cases, only 10% of cases are bilateral [12]. It occurs more frequently in full-term newborns and affects mainly males [7,13].

SARS-CoV-2 has a huge impact on newborns; neonates born from mothers with severe COVID-19 have a higher rate of prematurity, respiratory distress, moderate to severe hypoxic-ischemic encephalopathy, sepsis, and hyperbilirubinemia. Among those who died, 60% are due to severe hypoxic-ischemic encephalopathy with multi-organ dysfunction, and 40% were related to prematurity-related complications. In our case, the patient developed hypoxic-ischemic encephalopathy related to respiratory distress precipitated by severe maternal COVID-19, which ultimately may be considered a risk factor for AH [7].

Asphyxia at birth, sepsis, bleeding disorders, traumatic childbirth, and perinatal injuries, among others, are often considered risk factors for AH. A retrospective study identified vaginal delivery, macrosomia, and fetal acidemia as the most important risk factors for AH [13-14]. In our case, perinatal asphyxia, neonatal shock, and possibly severe maternal SARS-CoV-2 infection were risk factors associated with AH.

Neonatal adrenal hemorrhage related to SARS-CoV-2 has not been reported yet, thus there are no specific data related to the characteristics of AH related to COVID-19 in neonates, however, there have been cases among adults. Although the exact mechanism of adrenal hemorrhage is still unclear, it is thought that severe hyperinflammatory response and high levels of acute-response cytokines (TNF, IL-1β, IL-6, G-CSF) and chemotactic cytokines (IL-8 and MCP-1) related cytokine storm may play an important role. High levels of cytokines lead to endothelial dysfunction, vascular injury, and adrenal parenchymal damage leading to adrenal hemorrhage [15]. Furthermore, in high physiological stress, such as sepsis, adrenocorticotrophic hormone (ACTH) secretion increases, which stimulates blood flow to the adrenal glands. It exceeds the limited venous adrenal drainage, leading to a predominant venous hemorrhage [16].

AH in neonates presents with a wide range of clinical manifestations; the majority of patients are asymptomatic. The most common signs and symptoms include jaundice (in 50% of cases), ischemia, anemia, palpable abdominal mass, and scrotal hematoma in men. In severe cases, it could lead to adrenal insufficiency and even death [17].

When a newborn presents an adrenal mass, the differential diagnosis should include masses should include adrenal hemorrhage, adrenal cyst, adrenal abscess, neuroblastoma (NBL), and congenital adrenal hyperplasia, among others. NBL is the most important differential diagnosis, especially in unilateral cases, which was not the case for our patient [18].

In newborns, ultrasound is preferred for both initial screening and follow-up. Computed tomography and magnetic resonance imaging may be useful to confirm the diagnosis but usually do not provide additional information. A conservative approach is preferred; the patient should undergo imaging to assess hemorrhage resolution [2]. We decided to take a conservative approach in our patients with imaging and laboratory exams done periodically. The patient remained asymptomatic and a follow-up ultrasound showed complete hemorrhage resolution.

Gyurkovits Z et al. reported that out of 26,000 newborns who were examined via abdominal ultrasound, 74 infants (0.28 %) had AH, and only 1 had a bilateral adrenal hemorrhage; this patient developed AI. In contrast, our patient developed relative adrenal insufficiency without either hemodynamic dysregulations or biochemical laboratory exam alterations, that resolved after the acute event. AH should resolve within four weeks if there is no reduction in size or changes in echogenicity, complementary studies should be conducted to rule out malignancy [14].

Adrenal insufficiency (AI) occurs rarely even in bilateral adrenal hemorrhage due to incomplete impairment of the adrenal gland since only 10% of the adrenal gland is needed to prevent AI [12]. Furthermore, hemorrhage usually occurs in the subcapsular portion of the adrenal gland, without affecting the adrenal cortex [17].

Serum cortisol levels should be evaluated according to the clinical status of the newborn. A serum cortisol level within normal limits in a newborn under heavy stress does not exclude the diagnosis of adrenal insufficiency because, in these patients, normal cortisol may be indicative of relative adrenal insufficiency, normal cortisol may be relatively insufficient for heavy stress, as in the reported case [19].

## Conclusions

Neonatal adrenal hemorrhage should be primarily suspected in patients with significant risk factors. Maternal severe COVID-19 may be a contributing factor to AH. It is usually asymptomatic, but it could lead to relative adrenal insufficiency and even death; therefore, monitoring clinical status is crucial. A conservative approach is the gold standard, early surgical approach should be avoided. Follow-up should include imaging and laboratory examination to assess the resolution of adrenal hemorrhage and relative adrenal insufficiency.
